# Polymorphisms in *HIFs* and breast cancer sutarsceptibility in Chinese women: a case–control study

**DOI:** 10.1042/BSR20180950

**Published:** 2018-09-14

**Authors:** Changyou Shan, Yi Zheng, Meng Wang, Shuai Lin, Tian Tian, Yujiao Deng, Peng Xu, Qian Hao, Ying Wu, Tielin Yang, Yan Guo, Zhijun Dai

**Affiliations:** 1 Department of Oncology, The Second Affiliated Hospital of Xi’an Jiaotong University, Xi’an 710004, China; 2 School of Life Science and Technology, Xi’an Jiaotong University, Xi’an 710049, China

**Keywords:** breast cancer, case-control study, HIFs, polymorphism

## Abstract

Hypoxia-inducible factors (HIFs) play a crucial role in cancer progression. Several epidemiological studies have demonstrated that HIFs polymorphisms can influence the susceptibility of multiple cancers. However, the relationship of HIFs polymorphisms (rs11549467 and rs17039192) and breast cancer (BC) risk was still unknown. Thus, we performed a case-control study based on 560 BC patients and 583 healthy controls to explore the association between them. Our results indicated a boardline connection between HIF-1 rs11549467 and BC risk (AG compared with GG: OR = 1.61, 95% CI = 1.05–2.49, *P*=0.03; AG + AA compared with GG: OR = 1.64, 95% CI = 1.08–2.51, *P*=0.02; AG compared with GG + AA: OR = 1.61, 95% CI = 1.04–2.48, *P*=0.03; OR = 1.64, 95% CI = 1.09–2.45, *P*=0.02), while HIF-2 rs17039192 had no influence on breast cancer. Considered the comparison of sample size and potential heterogeneity of previous case–control studies, we concluded that HIF-1 rs11549467 has a marginal effect on BC risk. Further well-designed studies with larger sample size were required.

## Introduction

From the evidence supplied by *Cancer Statistics, 2018*, a total of 266, 120 new breast cancer (BC) cases and 40,920 BC-related deaths were expected to happen in American women in 2018 [1]. Like in most other countries, BC has possessed the highest incidence of all malignancies in Chinese women, for which nearly 268,600 new cases were estimated to occur in 2015 [2]. Besides, BC incidence rate in Chinese women has increased more than twice as fast as global average since 1900s, especially in urban areas [3]. As is well known, breast carcinogenesis comprises multiple and complicated processes, in which cellular adaption to hypoxia was identified as a crucial step for BC progression [4,5].

It has been a common finding that intratumoral hypoxia indicates poorer prognosis, higher metastasis risk, and lower sensibility to radiotherapy and chemotherapy for cancer patients [6–9]. Hypoxia-inducible factors (HIFs) were exactly the crucial oxygen-sensitive transcription factors to facilitate hypoxia adaption of cells through mediating function of the genes involving in angiogenesis, erythropoiesis, metabolic reprogramming, metastasis etc. [10]. HIFs are heterodimers formed by a stably expressed subunit HIF-1β and an oxygen-regulated subunit HIF-1α (encoded by HIF-1A) or HIF-2α (encoded by HIF-2A), and they controlled activities of over 1000 target genes [11]. Both HIF-1α and HIF-2α have been found overexpressing in various cancers, including BC [12,13]. And overexpression of HIF-1α/HIF-2α was generally associated with short-term survival and increased mortality in cancer cases [14].

To date, several epidemiological studies have demonstrated the influence of HIFs polymorphisms on multiple cancers susceptibility, such as lung, breast, pancreatic, gastric etc. [15] Thereinto, two missense mutation loci rs11549467 and rs11549465 on HIF-1A, and HIF-2A polymorphism rs17039192 were most frequently discussed. As yet, there was no epidemiological study exploring the association between the rs11549467 and rs17039192 C111A polymorphisms in HIFs and cancer risk among Chinese women. Because we previously performed a meta-analysis to investigate the role of rs11549465 in breast cancer progression and found no correlation between them [16], this case–control designed study is aimed to investigate the association between the two remaining HIFs polymorphisms (rs11549467 and rs17039192) and breast cancer risk in Northwest Chinese women.

## Materials and methods

### Study population

Breast cancer cases (*n*=560) were enrolled in the Department of Oncology, The Second Affiliated Hospital, Xi’an Jiaotong University, from January 2013 to October 2014. All BC patients were newly diagnosed using pathology and detailed immunohistochemical analysis, as was described previously [17–19]. Patients with history of malignant diseases or those who received preoperative chemotherapy or radiotherapy were excluded. The controls were randomly recruited from healthy females, who underwent annual physical examinations in the medical examination center of the same hospital during the same period. Controls (*n*=583) were matched to cases based on age (±5 years). All participants were Han Chinese women and supplied a written informed consent to provide us detailed data about self-administration. The present study was approved by the Institutional Review Board of Xi’an Jiaotong University (Xi’an, China).

### Genotyping assay

Samples of peripheral blood were collected into EDTA-coated tubes and conserved at −80°C. We used the Universal Genomic DNA Extraction Kit (version 3.0, TaKaRa Bio Inc., Japan) to extract genomic DNA from whole blood samples, according to the manufacturer’s instructions. DNA concentration was confirmed by the DU530 UV/VIS spectrophotometer (Beckman Instruments, Fullerton, CA, U.S.A.). For candidate SNPs in HIFs, we used data from HapMap database of Chinese population and finally elected two SNPs (rs11549467 in HIF-1A, rs17039192 in HIF-2A) included in our study. SNP genotyping was conducted with Sequenom MassARRAY RS1000 according to the standard protocol recommended by the manufacturer. The corresponding primers used for each SNP in our study are listed in Table 1. Sequenom Typer 4.0 Software (Sequenom, San Diego, CA, U.S.A.) was used to manage the data.

**Table 1:** Primers used for the present study.

SNP_ID	1st-PCRP	2nd-PCRP	UEP_SEQ
rs11549467	ACGTTGGATGTTGAGGACTTGCGCTTTCAG	ACGTTGGATGCTTCCAGTTACGTTCCTTCG	gtccCCATTAGAAAGCAGTTCC
rs17039192	ACGTTGGATGACACTGCCGAGGATTGTACG	ACGTTGGATGTTTACACTCGCGAGCGGAC	aggcCCGCCACACGGGTCCGGTG

### Statistical analysis

Fisher’s exact test was used to exam the Hardy–Weinberg equilibrium (HWE) among controls for each polymorphism. Two-sided Pearson’s chi-square tests were adopted to evaluate the differences in allelic frequencies for each SNP between patients and controls, and it was considered statistically significant if the *P* value less than 0.05. Five different genetic models were used to evaluate the association between SNPs and breast cancer risk (‘A’ and ‘a’ are used to represent the major and the minor alleles, respectively): the allele model (a compared with A); the co-dominant model (homozygote model: aa compared with AA; heterozygote model: Aa compared with AA); the recessive model (aa compared with AA + Aa); the dominant model (AA compared with Aa + aa); and the overdominant model (AA + aa compared with Aa). We employed SPSS software to access odds ratios (ORs) and 95% confidence intervals (CIs).

## Results

### Characteristics of the patients and controls

The present study contained 560 BC patients and 583 healthy controls, the characteristics of which were described in our previous studies [17–19], the detailed information was displayed in Supplementary Table S1. Sporadic breast cancer patient recruited in this case–control study was diagnosed by histopathology. The average age of cancers group was 49.09 ± 11.02. The control group was matched depending on age (0.612) and menopausal status (*P*=0.716). However, the body mass index (BMI) of control groups was significantly higher than BMI of patient groups (22.52 ± 2.84 compared with 22.95 ± 3.21; *P*=0.038); thus, all results were adjusted to BMI.

### Association between HIF polymorphisms and breast cancer risk

We genotyped the two SNPs (rs11549467 and rs17039192) in 1143 included subjects (560 cancer cases and 583 healthy control), and the genotyping success rates of the two SNPs were 100%. The genotype distribution of control groups accorded with HWE (*P*=0.1183 and 0.74 for rs11549467 and rs17039192, respectively) presented statistically significance between breast cancer patients and control subjects. Statistical analysis showed that rs11549467 had an increased influence on breast cancer risk in the codominant, dominant, overdominant, and allele model (AG compared with GG: OR = 1.61, 95% CI = 1.05–2.49, *P*=0.03; AG + AA compared with GG: OR = 1.64, 95% CI = 1.08–2.51, *P*=0.02; AG compared with GG + AA: OR = 1.61, 95% CI = 1.04–2.48, *P*=0.03; OR = 1.64, 95% CI = 1.09–2.45, *P*=0.02), while rs17039192 was not associated with breast cancer (Table 2).

**Table 2:** Genotype frequencies of HIF polymorphisms in cases and control

Model	Genotype	Case (560)	Control (583)	OR (95% CI)	*P*-value
**HIF-1α SNP: rs11549467 HWE: *P*=0.1183**					
Codominant	G/G	501 (89.5%)	544 (93.4%)	1	–
	A/G	55 (9.8%)	37 (6.3%)	1.61 (1.05–2.49)	**0.03**
	A/A	4 (0.7%)	2 (0.3%)	2.17 (0.40–11.91)	0.37
Dominant	G/G	501 (89.5%)	544 (93.4%)	1	-
	A/G–A/A	59 (10.5%)	39 (6.6%)	1.64 (1.08–2.51)	**0.02**
Recessive	G/G–A/G	556 (99.3%)	581 (99.7%)	1	-
	A/A	4 (0.7%)	2 (0.3%)	2.09 (0.38–11.46)	0.40
Overdominant	G/G–A/A	505 (90.2%)	546 (93.7%)	1	-
	A/G	55 (9.8%)	37 (6.3%)	1.61 (1.04–2.48)	**0.03**
Allele	G	1057 (94.4%)	1125 (96.5%)	1	-
	A	63 (5.6%)	41 (3.5%)	1.64 (1.09–2.45)	**0.02**
**HIF-2α SNP: rs17039192 HWE: *P*=0.4976**					
Codominant	C/C	542 (96.8%)	552 (94.7%)	1	-
	C/T	18 (3.2%)	31 (5.3%)	0.59 (0.33–1.07)	0.08
	T/T	0 (0%)	0 (0%)	NA	-
Allele	C	18 (1.6%)	31 (2.7%)	1	-
	T	1102 (98.4%)	1135 (97.3%)	0.60 (0.33–1.08)	0.09

### The results of meta-analysis for rs11549467 and breast cancer

Since boardline connection was found between HIF-1 rs11549467 and BC risk, we conducted a meta-analysis based on five articles including 898 BC cases and 751 healthy controls (Table 3). Pooled analysis indicated that G1790A polymorphism was not associated with the risk of breast cancer in all three comparisons (all *P*>0.05). In subgroup analyses, no significant association was found with the risk of breast cancer among Asians and in the hospital-based studies (all *P*>0.05).

**Table 3:** Characteristics of studies included in this meta-analysis.

Year	First author	Country	Ethnicity	Source of controls	Cases			Controls			Cases	Controls	HWE
					GG	GA	AA	GG	GA	AA			
2014	Sharma [24]	India	Asian	Hospital	200	0	0	200	0	0	200	200	NA
2013	Ribeiro [29]	Portugal	Caucasian	Hospital	96	0	0	74	0	0	96	72	NA
2009	Naidu [30]	Malaysia	Asian	Hospital	332	72	6	222	50	3	410	275	0.92
2008	Apaydin [23]	Turkey	Caucasian	Population	102	0	0	98	4	0	102	102	0.84
2008	Kim [31]	Korea	Asian	Hospital	87	3	0	92	7	1	90	102	0.06

## Discussion

HIFs play a critical role in facilitating cells to adapt to hypoxia. Existing evidences proved that HIFs took part in many pivotal aspects of cancer progression. In this case–control study, we aimed to investigate the association between HIFs polymorphisms and breast cancer risk among Northwest Chinese women. The results indicated a boardline connection between HIF-1 rs11549467 and BC risk, while HIF-2 rs17039192 had no influence on breast cancer.

HIF-1 rs11549467 is a missense mutation polymorphism leading to an amino acid of alanine substituted by threonine. This amino acid change may significantly enhance the transcription activity of HIF-1A and effect on the downstream target genes expression [20,21]. The overexpression of HIF-1α might sequentially lead to an increased cancer susceptibility and metastasis [22]. In the past few years, several molecular epidemiological studies were performed to explore the relationship between HIF-1 rs11549467 and breast cancer in different regions and ethnicities. However, no credible conclusion was obtained because of their small sample size (presented in Table 3). For instance, Apaydin et al. [23] conducted a case–control study based on 102 BC cases and 102 healthy controls to access the correlation of rs11549467 and BC. There are 98 GG type and 4 GA type in 102 healthy controls and 102 GG type in 102 BC patients. Genotype frequencies of rs11549467 in their study present no statistical significance between BC patients and healthy controls. Another Indian study performed by Sharma genotyped rs11549467 among 200 breast cancers and 200 healthy controls, the minor allele frequency was zero [24]. Considered the sample size of single study was excessively small, we pooled previous relative case–control studies together as a meta-analysis. Based on five articles including 898 BC cases and 751 healthy controls, our meta-analysis demonstrated no association between rs11549467 and BC risk. In view of the potential heterogeneity may exist in this meta-analysis, the consequence of our case–control study seemed more authentic and reliable.

Rs17039192 is a functional polymorphism in HIF-2α, which was encoded by the human EPAS1 (for endothelial PAS domain protein 1) gene [25]. Previous studies found that HIF-2α expression was associated with BC patients prognosis [13,26]. Giatromanolaki et al. [27] reported that HIF-2α may induce angiogenesis in breast carcinomas. Compared with HIF-1α, the role of HIF-2α in solid tumors was less known. According to the study performed by Nakajima et al. [25], rs17039192 in HIF-2α was only reported to be connected with knee osteoarthritis. This is the first case–control study aimed to investigate the relationship between HIF-2α and breast cancer. The results suggested HIF-2 rs17039192 have no influence on breast cancer risk.

Breast cancer is a multiple-factorial disease with complicated etiopathogenesis, genetic factors only occupy a part of these. Focusing oncology research on genetic factors, 70 SNPs were identified as BC-related by large-scale replication studies and genome wide association studies. As a focus area of oncological research into genetic factors, 70 SNPs were identified as BC-related mutation by large-scale replication studies and genome wide association [28]. SNPs may influence cancer in many aspects involving susceptibility, diagnosis, treatment, and prognosis. Since the phenotypic effect of SNPs was depended on complex interaction of gene–gene or/and gene–environment, we failed to obtain a certain conclusion about the association between HIFs polymorphisms (rs11549467 and rs17039192) and breast cancer. Further well-designed studies with larger sample size were required.

The present study has some potential limitations. First, all the included subjects came from the same hospital name Xi’an Jiaotong University, thus selection bias may exist inevitably in the present study. Second, we failed to consider other significant risk factories such as lifestyle, living environment, and family history because of lack of data. Third, all analyses in our study were based on the genotype distribution of the two SNPs, functional studies revealed the molecular mechanism were absent.

In conclusion, our results suggest that HIF-1 rs11549467 has a marginal effect on BC risk, while no association was found between HIF-2 rs17039192 and BC risk.

## Supporting information

**Supplementary Table S1:** The characteristics of breast cancer cases and cancer-free controls.

## Abbreviations

BC: breast cancer
BMI: body mass index
CI: confidence interval
EPAS1: endothelial PAS domain protein 1
HIF: hypoxia-inducible factor
HWE: Hardy-Weinberg equilibrium
OR: odds ratio
