# Correction: Polymorphisms in HIFs and breast cancer susceptibility in chinese women: a case-control study

**DOI:** 10.1042/BSR-2018-0950_COR

**Published:** 2024-08-09

**Keywords:** breast cancer, case-control study, HIFs, polymorphism

This article is being corrected following notification from a reader alerting *Bioscience Reports* to a typographical error in the title of this paper, where “susceptibility” has been misspelled as “sutarsceptibility”. Following investigation by the Editorial Office, it was identified that the version of the manuscript accepted for publication on 20th August 2018 did not contain this spelling mistake, and that this error appears to have been introduced during the proofing stage of the production process. The Journal apologises for this error. The authors agree to the correction.

The title has been corrected to: “Polymorphisms in *HIFs* and breast cancer susceptibility in Chinese women: a case-control study”.

