# Supplementary material for: Polymorphisms in HIFs and breast cancer sutarsceptibility in Chinese women: a case–control study
Source: Biosci Rep. 2018 Sep 14;38(5):BSR20180950. doi: 10.1042/BSR20180950 (PMC6137243; doi:10.1042/BSR20180950)
Supplement: Supplementary file 1 [file bsr20180950_Supp1.pdf]

Supplementary Table S1 The characteristics of breast cancer cases and cancer-free controls.

Characteristics		Cases	Controls	*P*
Number		560	583	
Age (years, mean ± SD)		49.09±11.02	48.80±8.28	0.612
< 49		294	311	
≧49		266	272	
Menopausal status				
Premenopausal		264	281	
Postmenopausal		296	302	0.716
Procreative times				
<2		289	291	0.594
≥2		271	292	
Body mass index (kg/m^2)				
(mean ± SD)		22.52±2.84	22.95±3.21	0.038
Tumor size	<2 cm	188		
	≥2 cm	372		
LN metastasis	Negative	236		
	Positive	324		
ER	Negative	247		
	Positive	313		
PR	Negative	255		
	Positive	305		
Her-2	Negative	389		
	Positive	171		
Ki67	< 14%	195		
	≥ 14%	365		
